# Radiotherapy access in Latin America: Socio-economic determinants and equity challenges socio-economic determinants in Latin America for radiotherapy

**DOI:** 10.1016/j.ctro.2025.101062

**Published:** 2025-10-27

**Authors:** Gustavo R. Sarria, Santiago Torales, Florencia Rossi, Leandro Ricagni, Dante Baldeon, Armando Felix, Benjamin Li, Eleni Gkika, Gustavo Ferraris, Gustavo J. Sarria

**Affiliations:** a Department of Radiation Oncology, University Hospital Bonn, University of Bonn, Bonn, Germany; b Agencia de Evaluacion de Tecnologias Sanitarias de Uruguay (AETSU), Montevideo, Uruguay; c Department of Radiation Oncology, Hospital de Clinicas, Montevideo, Uruguay; d Department of Cancer Control & Prevention, Instituto Nacional de Enfermedades Neoplasicas, Lima, Peru; e Department of Radiotherapy, Hospital Angeles Lomas, Mexico City, Mexico; f Rayos Contra Cancer Inc, Seattle, WA, USA; g Division of Radiation Oncology, Fred Hutch Cancer Center, University of Washington, Seattle, WA, USA; h Radiotherapy Unit, Centro de Radioterapia Dean Funes, Cordoba, Argentina; i Department of Radiation Oncology, Oncosalud-Auna, Lima, Peru; j Department of Radiotherapy, Instituto Nacional de Enfermedades Neoplasicas, Lima, Peru

**Keywords:** Radiotherapy, Latin America, Socio-economic determinants, Access, Disparities

## Abstract

**Introduction:**

Radiotherapy (RT) is essential for cancer treatment, yet access in Latin America remains highly unequal due to socio-economic and systemic disparities. This study aims to identify and analyze the key socio-economic determinants influencing RT access, infrastructure, and workforce distribution across 11 Latin American countries.

**Methods:**

A comprehensive database was created using 29 demographic, economic, and healthcare-related variables from public sources and expert input. Countries included were Argentina, Bolivia, Brazil, Chile, Colombia, Ecuador, Mexico, Paraguay, Peru, Uruguay, and Venezuela. Variables were categorized under access, demand, and supply of RT services. Correlation analyses and linear/exponential regression models were applied in an exploratory way to evaluate relationships between socio-economic indicators and RT availability**.**

**Results:**

Higher GDP per capita and adjusted GDP (PPP) correlated significantly with better RT infrastructure, including EBRT and megavoltage units (r > 0.68, p < 0.05). Urban population percentage strongly correlated with RT access (r = -0.77, p = 0.005), while social security coverage was linked to lower inhabitants per RT center (r = -0.67, p = 0.025). Notably, the number of radiation oncologists correlated perfectly with patients requiring EBRT (r = 1.0, p < 0.001), but showed no correlation with poverty or urbanization, highlighting workforce capacity constraints. Rural areas were underserved due to infrastructure centralization in urban zones. High out-of-pocket expenditure and low public health investment would be associated with limited access to these treatments.

**Conclusion:**

Socio-economic disparities—particularly GDP, healthcare coverage, and urbanization—are strongly associated with RT access inequities in Latin America. For the medical community and public policymakers, confirming these assumptions requires a different scope about discussions regarding access to these highly complex services, when they are not associated with the population’s health needs but rather with the countries’ mere organizational and financial capabilities. Some possible formats require the definition of new clinical and financial management models. Our findings underscore the need for targeted health policies, investment in infrastructure and workforce, and decentralized care models. Expanding RT services beyond urban centers and improving funding models are critical to ensuring equitable cancer treatment across the region.

## Highlights

• First socio-economic analysis on radiotherapy access in the region.
• RT access in Latin America is highly unequal across countries.
• GDP and urbanization strongly influence RT access.
• Policy changes needed for equitable cancer care access.

## Introduction

Radiotherapy is a vital treatment for cancer patients, significantly influencing survival rates. Delays in treatments could even impair survival outcomes [1,2]. However, ensuring universal and equitable access remains a challenge, particularly in low- and middle-income countries (LMICs), where healthcare coverage is often limited [3]. Cost-effectiveness concerns, while important, should not solely dictate the availability of critical cancer treatments. Instead, policies should focus on health equity and rational resource allocation, especially in regions where market-driven distribution models often fail to cover public health needs. However, more quantitative data is needed to guide and promote an equitable allocation of resources.

In general, macroeconomic and social determinants play a fundamental role in shaping healthcare access. Key factors include national wealth, overall development, and healthcare spending as a percentage of Gross Domestic Product (GDP). A considerable number of complex variables, including medical and technical criteria, as well as the economic, political, cultural and organizational framework at the level of health systems, influence the supply and demand for healthcare services [4]. These interactions lead to asymmetries in healthcare accessibility that become more evident when comparing countries with different levels of economic development, a situation clearly represented in Latin America. Political decisions, financing models, infrastructure and technology conditions, availability of labor and medical supplies, as well as socio-economic inequalities and geographic characteristics are varied in the region and particularly impact healthcare opportunities and outcomes [5].

Latin America, with 630 million people (9 % of the world's population) and up to 80 % residing in cities, is the most urbanized region on the planet. Despite needing to care for a more aged population, with the life expectancy four years higher than the world average (76 versus 72 years), only an average of 6 % of GDP is spent on healthcare, lower than the 8.8 % reported by countries in the Organization for Economic Cooperation and Development (OECD) [6,7]. Likewise, the region faces severe disparities and inequities: approximately 250 million people (46 % of the population) lack social security, 54.2 million households (39 % of households) depend exclusively on informal employment, and poverty rates are at 29.2 %, with 11.2 % in extreme poverty [8]. These factors set the context for challenges faced in cancer care.

Ensuring equitable access to radiotherapy in Latin America is a multifaceted challenge shaped by many of the aforementioned socio-economic determinants. Access remains highly unequal with significant disparities between urban and rural areas, public and private healthcare systems, and high- and low-income populations [9] .Many countries face insufficient infrastructure, a shortage of radiation oncologists, outdated equipment, and inadequate funding, limiting availability [10]. According to various analyses, several Latin American nations lack the necessary number of radiotherapy units to meet patient demand, leading to long waiting times and delayed treatments. Addressing these gaps requires increased investment, policy reforms, regional collaboration, and innovative funding models to ensure equitable access to life-saving cancer treatment [5,11,12].

In order to enable the development of targeted healthcare policies that can improve access to treatment, we must first identify and characterize the potential root sources of inequity. In this context, this study aims to analyze in an exploratory and ecological sense the impact and grouping of various socio-economic determinants on the availability and access to radiotherapy services across Latin America in order to establish possible associations regarding resource allocation criteria in contrast to population health needs.

## Materials and methods

First, we considered multiple preliminary relevant socio-economic, demographic, and healthcare-related variables to study. The selection of demographic variables (total population and urban concentration), socio-economic variables (wealth/poverty and equity measurements), healthcare systems variables (expenditure and coverage), and outcome variables were obtained from previous publications, yielding 29 total variables [8]. Then, a preliminary database was created that included 11 Latin American countries (Argentina, Bolivia, Brazil, Chile, Colombia, Ecuador, Mexico, Paraguay, Peru, Uruguay, and Venezuela). For each country, we included these variables using data from multiple published sources [5,6,[13], [14], [15], [16], [17], [18], [19], [20]].

Next, we refined and narrowed the list of variables. We considered the validity of the demographic and socio-economic variables to reflect the particular conditions of the countries in a traceable way and the application of similar approaches in the analyses of other highly complex practices [8,14,20]. We adjusted the outcome variables by consensus with radiotherapy experts in the region, sorting them into three categories: access, demand and supply. We finalized our selection of all variables according to the availability, reliability and timeliness of the data sources, as well as the consensus of applicability with the participating experts. The final variables (Table 1) and Table 2 display a subset of the sociodemographic and economic variables for each country with available data of radiotherapy needs and services.

**Table 1:** List of socio-economic variables and sources selected for the analysis.

Variables	Source
ACCESS	
1. Inhabitants/EBRT center	Sarria et al. 2023
DEMAND	
1. Patients requiring EBRT (PMP)	Sarria et al. 2023
2. Patients requiring brachytherapy (PMP)	Sarria et al. 2023
SUPPLY	
1. RT Centers (PMP)	DIRAC, 2025
2. Brachytherapy units (PMP)	DIRAC, 2025
3. Megavoltage RT units (PMP)	DIRAC, 2025
4. Kilovoltage RT units (PMP)	DIRAC, 2025
5. Total RT Units (PMP)	DIRAC, 2025
6. Radiation oncologists (PMP)	Sarria et al. 2023
7. Medical Physicists (PMP)	Sarria et al. 2023
8. Radiation Therapists (PMP)	Sarria et al. 2023
9. Year of first commissioning (first RT treatment historically)	Pinillos et al. 2017
DEMOGRAPHICS	
1. Total population (inhabitants)	CEPAL, 2019
2. Urban population (%)	CEPAL, 2019
3. Population density (inhabitants/km^2^)	CEPAL, 2019
ECONOMICS	
1. GDP (US$)	PAHO-WHO, 2019
2. GDP adjusted by purchasing power parity (US$)	PAHO-WHO, 2019
3. World Bank Classification	World Bank, 2020
4. Gini Index	PAHO-WHO, 2019
5. Kuznets Ratio	PAHO-WHO, 2019
6. Poverty rate (%)	PAHO-WHO, 2020
7. Extreme poverty rate (%)	PAHO-WHO, 2020
HEALTHCARE SYSTEM	
1. Total health expenditure (%)	PAHO-WHO, 2019
2. Private health expenditure (%)	PAHO-WHO, 2019
3. Public health expenditure (%)	PAHO-WHO, 2019
4. Out-of-pocket health expenditure (%)	PAHO-WHO, 2015
5. Social security coverage (% population)	Torales et al. 2025
6. Public healthcare coverage (% population)	Torales et al. 2025

RT: Radiotherapy; EBRT: external-beam radiotherapy; PMP: per million population; GDP: gross domestic product; DIRAC: Directory of Radiotherapy Centers (International Atomic Energy Agency); ECLAC: Economic Commission for Latin America and the Caribbean (United Nations); PAHO-WHO: Pan American Health Organization – World Health Organization; SLANH: Latin American Society for Nephrology and Hypertension

**Table 2:** Subset of the sociodemographic, economic and radiotherapy variables.

Country	Population	Urban population (%)	GDP per capita(US$)	Health expenditure (% GDP)	Out of pocket health costs	Poverty rate (%)	EBRT Center	Patients requiring brachytherapy	Radiation oncologists	Medical physicists	Radiation therapists	Starting year RT programs
Argentina	44.745.517	92,3	9.947	9,8	17,6	27,2	117	65,936	264	137	380	1930
Bolivia	11.777.312	69,9	3.552	7,2	22,5	31,1	8	10,650	43	9	38	1941
Brasil	211.782.880	86,7	8.898	10,1	20,2	19,2	272	316,170	1265	450	2000	1897
Chile	19.039.490	88	14.742	9,2	35,4	10,7	45	20,778	83	51	143	1930
Colombia	50.187.403	77,3	6.425	8,2	18,3	31,7	70	59,749	239	104	309	1919
Ecuador	17.343.737	64,3	6.223	8,4	41,6	25,7	23	16,647	67	22	72	1954
México	125.085.315	78,2	9.946	5,5	40,8	41,5	145	116,986	468	180	250	1903
Paraguay	6.530.027	68,2	5.415	7,2	31	19,4	5	10,878	44	4	15	1943
Perú	32.824.866	83,3	7.097	5,4	30,9	15,4	32	34,220	137	40	112	1925
Uruguay	3.428.407	96,5	17.768	9,6	16,2	3	13	5750	23	6	70	1922
Venezuela	28.971.679	89,2	6.313	5,1	28,2	20,3	87	36,435	146	172	411	1929

GDP: gross domestic product; EBRT: external-beam radiotherapy; RT: Radiotherapy.

Finally, our exploratory analysis of these variables in our constructed database focused on determining in what way or magnitude the socio-economic determinants influence the access, demand and availability of radiotherapy services in Latin America. The Pearson correlation coefficient (r) was used to assess the relationship between all selected variables. Statistical significance was defined as p < 0.05 in all cases. Variables that demonstrated statistically significant correlations were further analyzed using univariate linear or exponential regression, according to the best fit. The most relevant regression models (R^2^ ≥ 0.6) are presented as figures, and only statistically significant correlations are reported in the tables. Residual normality was assessed through visual inspection of residual plots against the independent variable. All analyses were conducted using JASP (JASP V0.19, JASP Team 2024 [Computer software], Amsterdam, The Netherlands).

## Results

**Fig. 1:**
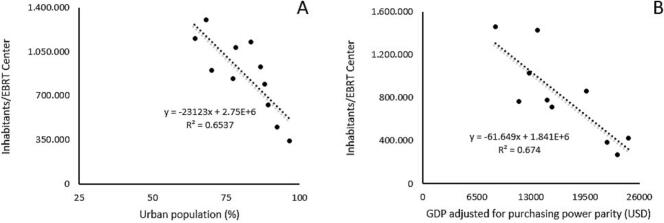
Linear regression assessment of Inhabitants per EBRT Center and their correlation to urban population density **(A)** and GDP adjusted for purchasing power parity **(B)**. A tendency towards higher concentration of facilities in urban areas is noticeable.

In total, 364 correlations were performed. Only those that were statistically significant are displayed in tables. Access, demand, and the availability of radiotherapy services and professionals were correlated, with main findings summarized in Table 3, using different correlations for variables related to radiotherapy access (as population density per EBRT center). The number of patients requiring brachytherapy exhibited a negative correlation with GDP per capita (p = 0.014) and GDP adjusted for purchasing power parity (p = 0.018). Fig. 1 illustrates the best-fitting linear regression models (R^2^ ≥ 0.60) between variables that were tested for their relationship to radiotherapy access.

**Table 3:** Correlation between radiotherapy variables and others.

	Inhabitants/EBRT Center r (p value)	Patients requiring brachytherapy (PMP) r (p value)	Radiation oncologists (PMP)	Medical physicists (PMP)	Radiation therapists (PMP)	Starting year RT programs
Total population	NS	NS	NS	NS	NS	−0.821 (0,002)
Urban population	−0,774 (0,005)	NS	NS	NS	0,768 (0,006)	NS
GDP per capita	−0,687 (0,020)	−0,710 (0,014)	NS	NS	NS	NS
GDP adjusted by purchasing power parity (US$)	−0,821 (0,004)	−0,722 (0,018)	NS	0,731 (0,016)	NS	NS
Poverty rate (%)	NS	NS	NS	NS	−0.639 (0,034)	NS
Population covered by social security	−0,668 (0,025)	NS	NS	0,612 (0,046)	NS	NS
Population per center	NS	NS	NS	NS	−0.838 (0,001)	NS
RT Centers (PMP)	−0,696 (0,017)	NS	NS	NS	0.863 (<0,001)	NS
Megavoltage RT units (PMP)	−0,624 (0,040)	NS	NS	NS	NS	NS
Brachytherapy units (PMP)	NS	NS	NS	NS	0.763 (0,006)	NS
Total RT Units (PMP)	NS	NS	NS	NS	0.806 (0,003)	NS
Medical Physicists (PMP)	−0,696 (0,017)	NS	NS	−	NS	NS
Radiation Therapists (PMP)	−0,755 (0,007)	NS	NS	NS	−	NS
Patients requiring brachytherapy (PMP)	0,746 (0,008)	−	NS	NS	NS	NS
Patients requiring EBRT (PMP)	NS	NS	1,000 (<0,001)	NS	NS	NS

RT: Radiotherapy; EBRT: external-beam radiotherapy; GDP: gross domestic product; PMP: per million population; NS: not significant.

**Fig. 2:**
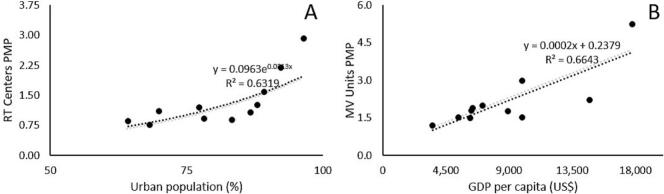
Linear regression of density of EBRT centers and MV units per one million inhabitants and its correlation with urban population density **(A)** and GDP adjusted for purchasing power parity **(B)**. PMP: per million population.

Table 3 presents the statistically significant correlations for variables related to the demand for EBRT and brachytherapy. Furthermore, a positive relationship between the number of EBRT centers per one million inhabitants and the percentage of urban population was modeled with linear regression. Similarly, a linear regression model showed the positive relationship between the number of megavoltage units per one million inhabitants and GDP per capita. These are both shown in Fig. 2**.**

Regarding the availability of radiotherapy professionals and other analyzed factors, the number of radiation oncologists showed no correlation with any sociodemographic variable but exhibited a perfect positive Pearson correlation with the number of patients requiring EBRT.

**Fig. 3:**
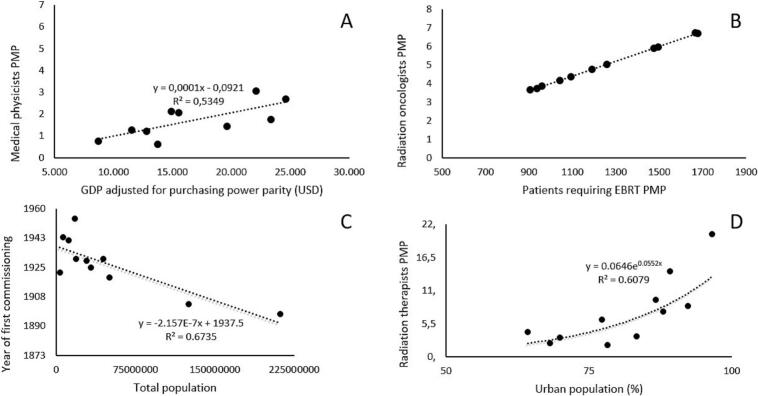
Both linear and exponential regression models for the strongest correlations between medical physicists PMP and GDP adjusted for purchasing power parity **(A)**, radiation oncologists PMP and patients requiring EBRT PMP **(B)**, year of first commissioning and total population **(C)**, and radiation therapists PMP and urban population density **(D).**

Furthermore, the following correlations were observed**:** the availability of medical physicists showed a significant positive correlation with GDP adjusted for purchasing power parity (p = 0.016) and the population covered by social security (p = 0.046). The commissioning year demonstrated a negative correlation with the total population (p < 0.001). Additionally, the number of radiation therapists correlated positively with urban population (p = 0.006), RT centers (p < 0.001), brachytherapy units (p = 0.006), megavoltage units (p = 0.003), and total radiotherapy units (p = 0.003), while showing a negative correlation with the poverty rate (p = 0.034). In Fig. 3, both linear and exponential regression models illustrate the availability of services in relation to the best-fitting sociodemographic variables per each dependent variable.

## Discussion

This study explores for the first time how economic disparities, healthcare investment, workforce distribution, and geographical barriers influence the availability and utilization of radiotherapy services across Latin America. The study was performed based on reports elaborated with first-hand information, obtained directly from local sources and national societies, which translates into reliable, up-to-date data. Associations between GDP, urbanization, the radiotherapy workforce, and radiotherapy availability were all found and characterized for the first time in the region. The correlations observed between key variables highlight the urgent need for policy interventions to bridge existing gaps in access to life-saving cancer treatment.

Countries with higher GDP per capita and adjusted GDP for purchasing power parity (PPP) tended to have better infrastructure, including a greater number of radiotherapy centers and brachytherapy units per million people (PMP). This is consistent with global patterns, where increased healthcare expenditure as a percentage of GDP is associated with greater access to specialized cancer treatments, including radiotherapy. However, Latin America lags behind the OECD standard, with an average healthcare expenditure of 6 % of GDP compared to 8.8 % in OECD countries. This discrepancy suggests that even middle-income countries in the region may struggle to allocate sufficient resources to ensure equitable access to cancer care.

In countries where patients bear a larger share of healthcare costs, access to radiotherapy was more restricted. This suggests that a significant barrier to radiotherapy access is the high level of out-of-pocket health expenditures. This relationship underscores the importance of publicly funded healthcare models and universal coverage policies in reducing financial barriers, particularly for lower-income populations, as countries with robust social security systems tend to report lower out-of-pocket costs and higher densities of radiotherapy units.

Our data also showed disparities in the availability of specialized personnel, including radiation oncologists, medical physicists, and radiation therapists. Unlike infrastructure, the workforce distribution did not appear to correlate with traditional sociodemographic factors, such as poverty. This indicates that systemic constraints, such as training capacity, workforce migration, and retention policies, may play a more significant role.

On one hand, a direct correlation existed between the number of medical physicists and radiation therapists per million people and GDP per capita, as well as the percentage of the population covered by social security. On the other hand, there was a lack of correlation with radiation oncologists. Nevertheless, this does not necessarily mean better occupancy of positions, because the lacking infrastructure drives practitioners to practice in other disciplines. Meanwhile, the almost perfect linear association between the number of radiation oncologists and the number of patients per country would seem to be due to a full quota effect or capacity limit related to the working hours of specialists or scheduling from centers.

Finally, the correlation between radiotherapy service implementation and population density indicates that longstanding infrastructure is more prevalent in highly populated areas, while rural regions remain underserved. The strong correlation between urbanization and radiotherapy availability reinforces previous findings that more than 70 % of Latin American radiotherapy centers are located in major metropolitan areas, leaving rural populations with limited access to treatment options. This reflects historical investment patterns in urban centers, where healthcare facilities and trained professionals are concentrated. Furthermore, the negative correlation between the first year of commissioning and population denote a trend towards increased development in highly populated areas, with an early onset during the first years of the 20th century. However, this urban-centric approach has left significant gaps in access for patients residing in rural and remote areas. Patients in rural communities face substantial obstacles, including long travel distances, financial constraints, and logistical challenges. These barriers contribute to lower adherence rates to prescribed radiotherapy regimens, which can lead to poorer cancer outcomes and should be seriously considered by both providers and policy makers.

Considering these findings, we believe that healthcare discussions could be approached from a different perspective: if we fail to recognize this clear decision-making framework for resource allocation, we are likely generating proposals incorrectly and expecting solutions where none exists. This potential gap between the expectations of physicians and their patients (albeit explicit, defensible, and even data- and evidence-based) and real possibilities or executive plans from political actors must be addressed with new perspectives for unrolling investments. One possible point for reconsidering access to high complexity care will involve the evaluation of models based on supply subsidies with a commitment to monitoring their quality and efficiency. Opting for the establishment of private centers under competitive schemes and demand subsidies (in theory more efficient) would be an attractive alternative. Nonetheless, these require greater clinical resources, as well as innovative contractual and accountability mechanisms from government healthcare financing agencies.

This study presents some shortcomings. Despite the good first-hand quality of data, certain centers, mostly peripheral or rather small, might have not be considered within the data collection. Precise information on personnel is quite challenging, as this is a constantly changing panorama. Deeper details on outdated technology was not considered, as this data were not widely available. The heterogeneity of health care systems and investment profiles in the region does not allow elaborating specific recommendations. Country-based solutions should be tailored according to each own panorama. This work is not intended to serve the purposes of health planning, but rather is aimed at a general review of the socioeconomic context for the use of resources in response to explicit health needs. In the case of tertiary centers, this would be largely determined by the organizational and financing characteristics of the countries, thus explicitly generating inequity.

The sources of information have been validated and published from official statistics. Even so, data closest and prior the COVID-19 pandemic were adjusted for proper comparison. The pandemic disrupted socioeconomic indicators in these countries as well as the access to RT treatments [21].

From a statistical viewpoint, the limited number of countries in the region poses a sampling limitation, preventing more comprehensive analyses through multivariate methodologies. Although correlations from selected variables were established, they do not imply causality or direct association (only in an exploratory sense), nor do they determine the relative weight of the most sensitive variables. Nonetheless, the approximate values derived from combining methods could indicate associations for future lines of research on this topic.

Finally, while there exists significant effort by scientific societies to consolidate RT registries, these efforts should be strengthened by considering the socioeconomic and historical context in which they are implemented. Regarding this, countries with large population, diverse healthcare systems and higher development of RT programs (Argentina, Brazil, Mexico) may have particular characteristics that might bias the analysis.

Taken together, this first analysis on socio economic determinants in access to radiotherapy presents relevant factors to consider during planning phases of increasing radiotherapy availability. Expanding radiotherapy services beyond major cities is a critical step toward achieving equitable access. Potential strategies include the establishment of satellite radiotherapy centers, which could provide lower-complexity treatments, the deployment of mobile treatment units, and the implementation of telemedicine-based support systems to facilitate consultations and follow-ups for patients in underserved regions. Decentralizing radiotherapy services can improve access for rural populations. Establishing regional treatment centers outside major metropolitan areas and leveraging telemedicine platforms for consultations and follow-up care can significantly reduce geographical barriers. Artificial intelligence could also help close the gaps in radiotherapy, by alleviating the load on certain tasks, such as contouring or planning. Moreover, telemedicine holds the potential for further reducing these deficiencies.

## Conclusion

Certain socio-economic determinants show a strong correlation with gaps in access to radiotherapy, and its logical distribution seems to obey to economic and social development and possibilities. These factors might assist decision makers to advocate for radiotherapy in the most urgent areas and populations with the greatest need based on widely recognized and available demographic, socio-economic, and health system variables thinking from the perspective of new managed models.

## CRediT authorship contribution statement

**Gustavo R. Sarria:** Conceptualization, Data curation, Formal analysis, Investigation, Writing – original draft, Writing – review & editing. **Santiago Torales:** Conceptualization, Data curation, Writing – original draft, Writing – review & editing. **Florencia Rossi:** Formal analysis, Investigation. **Leandro Ricagni:** Supervision, Visualization. **Dante Baldeon:** Formal analysis. **Armando Felix:** Supervision, Validation. **Benjamin Li:** Visualization, Validation. **Eleni Gkika:** Supervision. **Gustavo Ferraris:** Resources, Supervision. **Gustavo J. Sarria:** Writing – review & editing, Visualization, Validation.

## Funding

No funding was obtained for this investigation.

## Declaration of Competing Interest

The authors declare that they have no known competing financial interests or personal relationships that could have appeared to influence the work reported in this paper.
